# Editorial: Training and education in neurosurgery: Challenges and strategies for the next ten years

**DOI:** 10.3389/fsurg.2022.984208

**Published:** 2022-08-16

**Authors:** Daniele Bongetta, Cesare Zoia

**Affiliations:** ^1^ Neurosurgery Unit, ASST Fatebenefratelli Sacco, Milano, Italy; ^2^ Neurosurgery Unit, Fondazione IRCCS Policlinico San Matteo, Pavia, Italy

**Editorial on the Research Topic** Training and education in neurosurgery: Challenges and strategies for the next ten years by Zoia C, Chaurasia B and Bongetta D. (2022) Front. Surg. 9: 984208. doi: 10.3389/fsurg.2022.984208

Training and education are the foundation of progress in every field of science. Even more so in surgical specialties, in which a sort of craftsmanship component is implied. In this regard, Neurosurgery has always been blessed by a fertile master-apprentice approach since its beginnings with Harvey Cushing being a mentor to Walter Dandy, all the way through the foundation of the most famous residency programs after WWII. In turn, Sir William Osler, the inventor of residency itself, was a mentor to Harvey Cushing (who, among other accolades, won a Pulitzer prize for writing a biography on him!). In his own words, Sir William Osler famously expressed the need for a combined theoretical-practical approach in medical learning: “He who studies medicine without books sails an uncharted sea, but he who studies medicine without patients does not go to sea at all.” (1). Even though this predicament is more than 100 years old, we should all agree that it is still true nowadays. But how are we providing notions and practical knowledge to new generations right now, and what are going to be the advances in training and education in the next ten years?

## Challenges

Several challenges have emerged in the last few years. First of all, Neurosurgery is expanding at an exponential rate. The advances made in subspecialties (endoscope use, interventional neuroradiology, minimally invasive spinal surgery), all come with the burden of a learning curve to master the technologies and techniques applied. The era of an omnicompetent neurosurgeon is quickly coming to an end as future generations should be ever more sub-specialized. Hence, the need for specialized, accurate, reliable training simulation solutions is emerging (García Feijoo et al.). Strictly related to these issues is the necessity for a trainee to be exposed to a sufficient caseload to become a specialist in a given procedure.For example, in dealing with the well-known debate on vascular neurosurgery, how could future generations learn to operate complex aneurysm surgeries if the number of craniotomies is diminishing each and every year? Moreover, the next generation will be ever more under the judgment of an open-data, quality-driven consumer market. During their training and practice, neurosurgeons will increasingly be asked to prove the quality of both their training and clinical outcomes. Residency programs, too, will undergo such a judgment, hence validation, implementation, reliability, and cost-effectiveness issues of training strategies will also be challenges to address. Dealing with cost issues, in particular, the urgent need for neurosurgeons in several Low-to-Middle-Income Countries (LMIC) clashes with the scarcity of affordable, widespread educational resources. The recent pandemic limitations too, have limited the possibilities to access lectures, courses, and workshops (2). Lastly, on a pedagogical level, newer generations might have different learning abilities that should be accounted for. Albeit poorly debated in scientific literature, attention span might be an issue for late millennials to early gen Z (born between 1990 and 2010). Marketing and new media companies report that due to the constant audiovisual stimulation to which they are exposed since childhood, newer generations are the first to tune out a speaker when the content does not engage them (3). On the contrary, the majority being familiar with video gaming activities, younger students demonstrate faster learning curves in acquiring surgical skills in which hand-eye coordination is more challenged, like robotic surgery and endoscopy (4).

## Strategies

**Figure 1:**
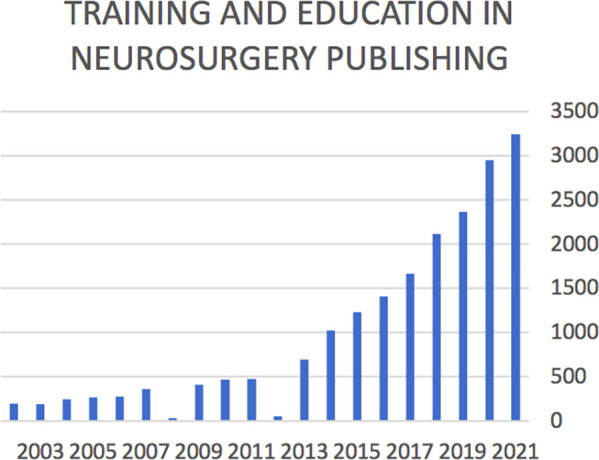
Number of publications about training and education in neurosurgery in the last two decades.

How is the neurosurgical community responding to these challenges? Albeit the limitations of such an analysis, querying PubMed database it appears that the number of publications about training and education in neurosurgery has grown steadily in the last two decades meaning an increasing interest (Figure 1).

Different approaches have emerged, all linked to increased use of technology which everyday becomes more available, powerful, and affordable (Calloni et al., Hanalioglu et al.).

On the training side, the improvement of computational power and the development of specific devices (eg Oculus and HoloLens) have led to the introduction of augmented or virtual reality (AR or VR) integration in surgical procedures (5, Cannizzaro et al). This not only has allowed the students to experience an unprecedented understanding of both normal and pathological anatomy, but it has also empowered neurosurgeons in rehearsing and simulating the different steps of a specific intervention (Turan Dundar et al.). The availability of 3D printing technology, moreover, has led to the possibility of hands-on training both on “home-made” models or on commercially available ones (6). This may be beneficial also in better preparing the students for well-established, classic, training tools such as cadaver dissections, whose access is limited by ethical, economic, and logistic constraints. Lastly, both virtual and 3D simulation tools may be soon employed as evaluation tools for residency programs, thus fostering both practical training and objective evaluation (Petrone et al.).

On the educational side, several digital tools have emerged in neurosurgery with free and low-cost mobile content. Nicolosi et al. have already outlined the potential advantages of such information sources (WFNS Young Neurosurgeons Forum Stream, Brainbook, NeuroMind, UpSurgeOn, The Neurosurgical Atlas, Touch surgery, The 100 UCLA Subjects in Neurosurgery, Neurosurgery Survival Guide, EANS Academy, Neurosurgical.TV, 3D Neuroanatomy, The Rhoton Collection, and Hinari) (7). In particular, they stress their optimal usability, with no time, space, device, or country of origin restraints. Indeed, these new digital tools may prove to be an excellent solution, especially for LMIC and considering the need for engaging, interactive, new teaching means for digital native students (Tiefenbach et al., Zoli et al.). The pandemic emergency limitations, furthermore, have boosted the organization of webinars on all topics of Neurosurgery. This has had obvious advantages, as webinars have been proven to be positively associated with achievement in knowledge, behavior, and skills. Nevertheless, the plethora of information provided has already been deemed potentially too overwhelming, calling for their better regulation by national and international scientific committees (8). Eventually, a new teaching trend is emerging in this hyper-technological era in which students have a tremendous wealth of information at their fingertips. Specifically, mentors should shift from the classical top-down teaching to a so-called “facilitating learning” method. The best teachers will be then those who will help students uncover and understand the information they find, with the ultimate goal of making them take ownership of their learning (9).

## Conclusions

The aim of this collection has been then to gather an up-to-date collection of high-quality original papers that could potentially inspire present and future masters and apprentices in our beloved craft of Neurosurgery. Moving from the excellent efforts collected, paraphrasing Sir William Osler, we might conclude that in the next ten years: “He who studies medicine without (books) technology sails an uncharted sea (or travels without Google maps!), but he who studies medicine without (patient) simulations firsts should not go to sea at all.”
